# Two Epidemiologic Patterns of *Norovirus* Outbreaks: Surveillance in England and Wales, 1992–2000

**DOI:** 10.3201/eid0901.020175

**Published:** 2003-01

**Authors:** Benjamin A Lopman, Goutam K Adak, Mark Reacher, David W.G. Brown

**Affiliations:** * Public Health Laboratory Service, London, United Kingdom

**Keywords:** Norwalk-like virus, Norovirus, Calicivirus, gastroenteritis, infectious intestinal disease, outbreaks, surveillance, research

## Abstract

In the period 1992–2000, the Public Health Laboratory Service Communicable Disease Surveillance Centre collected standardized epidemiologic data on 1,877 general outbreaks of *Norovirus* (formerly “Norwalk-like virus”) infection in England and Wales. Seventy-nine percent of general outbreaks occurred in health-care institutions, i.e., hospitals (40%) and residential-care facilities (39%). When compared with outbreaks in other settings, those in health-care institutions were unique in exhibiting a winter peak (p<0.0001); these outbreaks were also associated with significantly higher death rates and prolonged duration but were smaller in size and less likely to be foodborne. These data suggest that *Norovirus* infection has considerable impact on the health service and the vulnerable populations residing in institutions such as hospitals and residential homes. A distinct outbreak pattern in health-care institutions suggests a combination of host, virologic, and environmental factors that mediate these divergent epidemiologic patterns.

Recent population-based studies have shown that Noroviruses ([NVs] formal name: *Norovirus*; formerly “Norwalk-like viruses”) are the most commonly identified cause of infectious intestinal diseases in Western European communities (*1*,*2*). These viruses account for an estimated 6% and 11% of all infectious intestinal diseases in England and the Netherlands, respectively (*1*,*2*) and for an estimated 23 million cases of NV in the United States each year (*3*). NVs are also the most common cause of outbreaks of infectious intestinal diseases in Western Europe and North America (*3*–*7*).

Three factors contribute to the considerable impact of disease caused by NV: a large human reservoir of infection (*2*,*8*), a very low infectious dose (*9*), and the ability to be transmitted by a variety of routes. Person-to-person spread by means of the fecal-oral route or aerosol formation after projectile vomiting is the most commonly recognized mode of transmission (*4*,*10*), although foodborne (*3*,*11*) and waterborne (*12*–*14*) transmission are also well documented.

Gastroenteritis caused by NV is mild and self-limiting in the absence of other factors. Kaplan et al. and others have proposed that NV outbreaks can be recognized on clinical symptoms (short duration and incubation) and epidemiology (high attack rates and high frequency of vomiting) alone (*4*,*15*–*17*). Unlike rotavirus, NVs affect all age groups (*2*,*8*) The highest incidence is in children <5 years of age (*2*,*18*), but the greatest impact of NV is probably an economic one among the elderly in health-care institutions (*4*,*6*,*19*,*20*).

We describe the epidemiology of NVs in different outbreak settings. The data we present were collected by routine surveillance of general outbreaks of infectious intestinal diseases in England and Wales from 1992 to 2000 (*4*,*21*). Laboratory report surveillance of NV has been shown to be subject to a high degree of underascertainment (*8*) and age bias (*4*). Therefore, routine laboratory reporting of cases does not serve as a reliable sample for illness due to NV. For this reason, we describe only outbreak data.

## Methods

Since January 1992, the Public Health Laboratory Service Communicable Disease Surveillance Centre has operated a standardized comprehensive surveillance system for general outbreaks of infectious intestinal diseases (see Appendix). The details of how this system operates are described elsewhere (*4*,*21*). In 1995 and 1996, the Public Health Laboratory Service instituted an active reporting program for outbreaks of NV through the Electron Microscopy Network. Ten electron microscopy units, representing the principal regional diagnostic centers for viral gastroenteritis in England, reported to the Centre all general outbreaks for which clinical specimens had been submitted. These reports were then integrated into the existing outbreak surveillance system, and standardized epidemiologic data were sought from investigating public health physicians. The public health physicians contacted were asked to return completed questionnaires when investigations were concluded. Data from these questionnaires were entered and stored on an Epi Info 6.0 database (*23*).

### Statistical Analysis

We used the statistical software package STATA 6.0 for these analyses (*24*). Chi-square tests were used to compare proportions, and the Student t test was used to compare means. Data on persons affected and duration of outbreaks were observed to follow a non-normal distribution. Therefore, a natural log transformation was performed on the persons affected and duration of outbreak data to normalize the distribution of variables and satisfy the normality assumption for the t test (*25*). A reverse natural log transformation was then performed; results are presented as geometric means.

## Results

**Figure 1:**
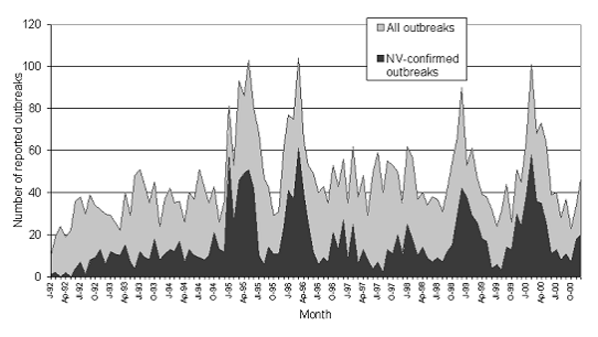
Seasonality of all outbreaks and confirmed *Norovirus* outbreaks, England and Wales, 1992–2000.

Completed outbreak questionnaires were returned for 5,241 general outbreaks occurring from January 1, 1992, to December 31, 2000 (response rate 73%). Laboratory confirmation of NV was recorded for 1,877 (36%) outbreaks (Figure 1). The median number of laboratory-confirmed cases in NV outbreaks was 2 (range 1–36). Another 731 outbreaks (14% of all outbreaks) were suspected of being caused by viral agents; 8 outbreaks were attributed to NV plus other pathogens; these outbreaks were excluded from these analyses.

### Settings

Information on setting was available for every NV outbreak (n=1,877). The most common settings were health-care institutions: 754 (40%) outbreaks occurred in hospitals and 724 (39%) in residential-care facilities. Information on the type of unit affected was available for 648 (86%) of 754 hospital outbreaks and 190 (26%) of the 724 in residential-care facilities. NV infection was centered on elderly care and geriatric units in 251 (39%) of 648 hospital outbreaks and 169 (89%) of 190 residential home outbreaks. A total of 147 (7.8%) outbreaks occurred in hotels, 73 (4%) occurred in schools, and 105 (6%) were linked to food outlets (Appendix). Seventy-four outbreaks (3.9%) occurred in other settings such as private homes, holiday camps, and military bases.

### Illness and Death

A total of 57,060 people were affected in the 1,877 NV outbreaks. After excluding hospital outbreaks (n=711), we recorded 128 hospitalizations (case-hospitalization rate = 33/10,000 cases) from 52 outbreaks (mean hospitalizations per outbreak 0.19; range 0–38). Forty-three deaths (case-fatality rate 7.5/10,000 cases) occurred in 38 outbreaks (mean deaths per outbreak 0.07; range 0–2); all were associated with outbreaks in hospitals (24 deaths) and residential-care facilities (19 deaths).

### Time Trends and Seasonality

**Figure 2:**
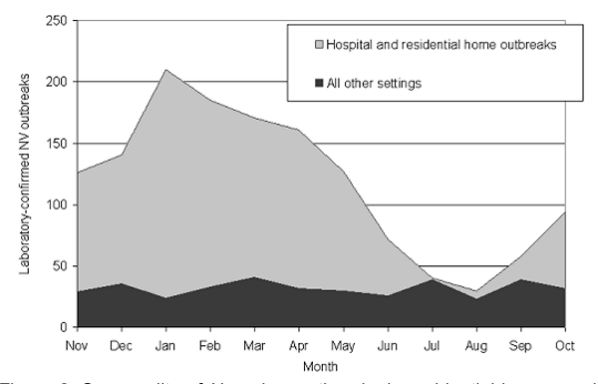
Seasonality of *Norovirus* outbreaks in residential homes and hospitals compared to all other settings, England and Wales, 1992–2000.

Reports of NV outbreaks peaked in 1995 (367 outbreaks) (Figure 1), falling to 139 outbreaks in 1997. Since then, outbreaks have steadily increased; 281 outbreaks were reported in 2000. Since 1995, outbreaks have shown a strong seasonal peak (Figure 1). Outbreaks begin increasing in September and peak in the months of January, February, and March. Outbreaks in hospitals and residential facilities occur more commonly in the 6 months from November to April than the rest of the year (994/421; ratio 2.36) (Figure 2). Outbreaks in other settings display no winter peak (189/205; ratio 0.92). This difference in the seasonality between outbreaks in health-care institutions and those in other settings is significant (χ^2^ 51.1, p<0.0001)

### Mode of Transmission

The reported modes of transmission were as follows (Table 1): person to person in 1,599 (85%) outbreaks; foodborne in 93 (5%) outbreaks; foodborne followed by person-to-person spread in 91 (5%) outbreaks; waterborne in 1 outbreak; unknown in 92 (5%) outbreaks.

**Table 1:** Primary modes of transmission of *Norovirus* outbreaks, England and Wales, 1992–2000

etting of outbreak	Foodborne^a^	Person to person^a^	Other/unknown^a^	Total
Hospital	10 (1.3)	716 (95.0)	28 (3.7)	754
Residential facilities	33 (4.5)	658 (91.0)	32 (4.4)	723
School	4 (5.5)	65 (89.0)	4 (5.5)	73
Food outlet	70 (66.7)	23 (21.9)	12 (11.4)	105
Hotel	42 (28.6)	94 (63.9)	11 (7.5)	147
Other	25 (33.8)	43 (58.1)	6 (8.1)	75
Total	184 (9.9)	1,599 (85.2)	93 (5.0)	1,877

^a^No. of outbreaks (% of all outbreaks in setting).

Person-to-person spread was reported in 716 (95%) of the 754 hospital outbreaks. This figure was a significantly higher proportion than observed in food outlets (22%; 23/105 [χ^2^ 551.3; p<0.0001], hotels (64%; 94/147 [χ^2^ 175.9; p<0.0001], schools (89%; 65/73 [χ^2^ 27.6; p<0.0001]), or residential facilities (91.0%; 658/723 [χ^2^ 13.9; p=0.0002]). Food outlets were the only setting where foodborne transmission predominated (67%; 70/105).

Person-to-person outbreaks occurred more commonly from November to April than in the rest of the year (1,020/514; ratio 1.98). Foodborne outbreaks showed a significantly weaker seasonality (105/73; ratio 1.43) than person-to-person outbreaks (χ^2^ 3.99; p=0.05).

### Food Vehicles

Specific vehicles were implicated in 72 (39.1%) of the 184 NV outbreaks reported to be transmitted by food. In 12 of these outbreaks, multiple food vehicles were reported, for a total of 86 implicated items. A wide range of food types were reported as vehicles of infection, including oysters, salad vegetables, poultry, red meat, fruit, soups, desserts, and savory snacks. The evidence implicating these food vehicles included cohort studies (55%; 47/86), case-control studies (8%; 7/86), and microbiologic studies (6%; 5/86) (Table 2). Reverse-transcriptase polymerase chain reaction (RT-PCR) was used to confirm viral contamination in oysters in all five outbreaks where microbiologic evidence was reported.

**Table 2:** Microbiologic and statistical evidence of foods implicated in outbreaks of *Norovirus*, England and Wales

Implicated food	Microbiologic evidence	Cohort study	Case-control study	Any evidence	Total no. of outbreaks in which food vehicle implicated
Oysters	5 (25%)^a^	9 (45%)	0	14(70%)	20
Poultry	0	6 (67%)	0	6 (67%)	9
Meat	0	3 (60%)	0	3 (60%)	5
Fish	0	3 (50%)	1 (16%)	4 (67%)	6
Salads and vegetables	0	10 (59%)	3 (18%)	13 (76%)	17
Other items	0	16 (55%)	3 (10%)	19 (65%)	29
Total	5 (6%)	47 (55%)	7 (8%)	59 (68%)	86

^a^Percentages represent outbreaks with evidence per total outbreaks where food vehicle was implicated**.**

### Contributory Factors

Contributory factors were reported in 113 (61%) of the 184 foodborne outbreaks. Infected food handlers were more commonly identified in food-related NV outbreaks (32%; 58/184) than in those caused by other pathogens (9%; 164/1750) (χ^2^ 80.39; p<0.0001). Contamination by an infected food handler was reported less frequently in outbreaks involving oysters than other foods (oysters 0%, other foods 47%; χ^2^ 14.69; p<0.0001). Cross-contamination was also reported less frequently in outbreaks involving oysters than other foods (oysters 5%, other foods 17%; χ^2^ 3.35; p=0.07).

### Duration

The median duration of outbreaks was 8 days (range 1–139 days). By setting, data on the duration of outbreaks were right-skewed since some outbreaks persisted for exceptionally long periods. The following results are therefore presented as geometric means. The duration of hospital outbreaks (8.8 days; 95% confidence intervals [CI] 8.4 to 9.3) was greater than those in food outlets (3.3 days; 95% CI 2.8 to 3.8; t = –12.699; p<0.0001) and hotels (4.3 days; 95% CI 3.6 to 5.1; t = –7.025; p<0.0001). However, the duration of hospital outbreaks and those in residential facilities did not differ significantly (8.7 days; 95% CI 8.1 to 9.4; t = –0.321; p=0.7) or schools (8.1 days; 95% CI 6.8 to 9.7; t = –0.879; p=0.4) (Table 3).

**Table 3:** Outbreak characteristics compared by setting of outbreak, England and Wales, 1992–2000

Setting	Median (days)	N	Geometric mean of duration (days)(95% CI)^a^	t test	p value
Duration of outbreaks					
Hospital	8	679	8.8 (8.4 to 9.3)		
Residential facilities	9	664	8.7 (8.1 to 9.4)	–0.321	0.73
School	8	63	8.1 (6.8 to 9.7)	–0.879	0.40
Food outlet	3	94	3.3 (2.8 to 3.8)	–12.699	<0.0001
Hotel	5	133	4.3 (3.6 to 5.1)	–7.025	<0.0001
Other	4	69	4.3 (3.6 to 5.1)	–8.043	<0.0001
All settings	8	1,702	7.7 (7.5 to 8.0)		
Numbers affected per outbreak					
Hospital	17	751	17.5 (16.4 to 18.5)		
Residential facilities	23	723	21.5 (19.8 to 23.3)	4.895	<0.0001
School	24	73	24.9 (20.5 to 30.3)	3.594	<0.0001
Food outlet	23	104	23.4 (19.8 to 27.6)	3.444	0.001
Hotel	29	147	26.5 (23.0 to 30.6)	5.729	<0.0001
Other	29	74	24.5 (20.2 to 29.7)	3.432	0.001
All settings	21	1,872	20.3 (19.7 to 21.1)		

^a^CI, confidence interval.

### Numbers of Persons Affected

The median number of persons affected per outbreak was 21 (range 2–1,200).

Data on the number of people affected in outbreaks were right-skewed since a number of outbreaks were exceptionally large. The following results are therefore presented as geometric means. The number affected in hospital outbreaks (17.5; 95% CI 16.4 to 18.5) was significantly lower than for other settings (geometric means 21.5 to 26.5; Table 3).

## Discussion

Examination of the features of NV outbreaks by setting reveals that outbreaks in health-care facilities have a distinctive epidemiologic profile. When compared with outbreaks in other settings, those in health-care institutions were unique in exhibiting a winter peak; they were also associated with higher death rates and prolonged duration but were smaller in size and were less likely to be foodborne. School outbreaks shared some but not all of the features that characterize outbreaks in health-care institutions.

Several epidemiologic and biologic reasons may contribute to the divergent seasonality. The respiratory infections season, which increases activity in health-care institutions, occurs concurrently with the peak in NV outbreaks in these facilities. Greater admission of patients in hospitals increases both the population at risk and the opportunities for NV to be introduced. An increase in transfers of people between residential-care facilities and hospitals also facilitates the movement of viruses between institutions. Populations in health-care facilities differ from the rest of the population in that they require nursing care. Health-care settings are semi-closed environments where patients and residents are subject to person-to-person spread and potentially contaminated environments.

Biologic differences between strains may also result in different clinical patterns. NVs from outbreaks in health-care institutions have less genetic diversity compared with those from other settings (*26*) or sporadic cases (*7*), and certain variants are more commonly found in health-care facilities than in other settings (*26*). Thus, the strong seasonality in health-care institutions may be the result of complex interaction between host, pathogen, and environment. If and how these factors contribute to the divergent patterns of health-care-associated and community outbreaks are unknown factors, but we believe that our findings warrant focused investigation in the UK and elsewhere.

The observation that a hospitalization was associated with 1 in every 40 outbreaks and a death with 1 in every 50 outbreaks calls into question the belief that NV gastroenteritis is a trivial disease. Although we have no information about the other health conditions of patients who were hospitalized or died, these figures are generated from laboratory-confirmed outbreaks. Previous estimates generated by Mead et al. (which were derived from Mounts et al.) were based on the assumption that NV causes a certain proportion of gastroenteritis hospitalizations and deaths (11%), an assumption that was not based on diagnostic results (*3*,*27*).

Deaths were only reported from outbreaks in health-care institutions. The populations in these institutions differ from those found in other settings by virtue of their greater age or presence of other underlying diseases. While NV infection is not likely the principal cause of death in most cases, this infection might constitute an additional burden on patients already weakened by other conditions and thus become an important contributory factor. In hospital outbreaks, attack rates among staff are similar to those among patients (*4*,*28*), suggesting that health status is not related to acquisition of disease but to severity of outcome. Therefore, efforts to control NV infection should be directed towards vulnerable persons who already require nursing care because of illness or injury.

The only settings in which foodborne transmission predominated were food outlets. That setting was the only category in which the purchase or consumption of food was the main factor linking at-risk populations. In other settings, living, working, or recreational areas were shared by at-risk populations for varying lengths of time, thus increasing the opportunities for person-to-person spread. Even in those instances where foodborne transmission initiated an outbreak within a health-care institution, high levels of person-to-person spread usually followed. Therefore, prolonged levels of contact between persons in semi-closed institutions such as hospitals, residential-care facilities, and schools facilitate person-to-person spread to an extent not seen in other settings, which in turn leads to more prolonged outbreaks. However, schools differ from health-care institutions in terms of the seasonality and duration of NV outbreaks. In this respect, schools are more like hotels, food outlets, and other settings.

The number of affected persons was smaller in hospital outbreaks than in all other settings. This finding may reflect the lack of a universally employed definition of the spatial boundaries of an outbreak. In some hospitals, each unit affected was reported as a separate outbreak, resulting in smaller but more numerous outbreaks. In addition, cases that occur in institutions are more easily recognized as part of an outbreak than cases in open settings or the community. Thus, smaller outbreaks occurring in open settings might not be recognized or reported to investigating agencies.

The peak in recorded outbreaks seen in the winter of 1995–1996 can largely be seen as a consequence of enhanced surveillance through the development of the Public Health Laboratory Service electron microscopy network. However, there are anecdotal reports of an increase in workload in these laboratories, and other countries also recorded an increase in NV activity during the same period (*11*). The steady increase of reports from 1998 to 2000 may be due to increased awareness, increasing use of the molecular diagnostics RT-PCR and enzyme immunoassays, or a real increase in the occurrence of outbreaks.

Biases in different surveillance systems partly explain the wide variation in estimates of the levels of foodborne transmission in NV outbreaks. The data presented in this report suggest foodborne transmission in 10% of outbreaks in England and Wales. Estimates in Sweden (16%) (*6*), the Netherlands (17%) (*11*), and the United States (40%) (*3*) were all higher; however, figures from these countries are derived from much smaller datasets. In the United States, foodborne outbreaks were more likely to be reported because surveillance may be focused on detecting foodborne outbreaks (*3*).

The data sources that contribute to a surveillance system are a key factor affecting the estimate of the importance of foodborne transmission. In England and Wales, surveillance is broad-based and collects reports on outbreaks spread by all modes of transmission from a range of public health professionals such as physicians, environmental health officers, and diagnostic laboratories. By contrast, FoodNet, a U.S. network, is designed to detect foodborne infections (*29*). Since hospitals in England and Wales are in the public sector, they might be expected to readily report outbreaks to the national surveillance scheme. However, by this logic, residential homes (which are privately operated) would not be expected to report outbreaks since they might be under commercial pressures to keep information on infection confidential. The fact that nearly as many outbreak reports came from residential homes as from hospitals in the survey period suggests that reporting predominantly from the public, not the private sector, is not the case. The biases on a passive surveillance system are multiple and cannot be expected to act in only one direction.

The importance of NV as a cause of gastroenteritis outbreaks in U.S. nursing homes has been demonstrated by Green et al. (*30*), although the role of this virus in hospital settings has not. Aside from bias, other reasons such as variability in infection control practices in different health-care systems could result in a real difference in the importance of foodborne transmission or transmission in health-care facilities. Although NV has been estimated to cause 67% of all such illness caused by identified microbial agents (*3*), only 5% of public health professionals considered this pathogen to be “one of the three most common pathogens causing foodborne illness in the United States” (*31*); this lack of awareness probably affects outbreak investigation.

The link between oysters and NV infection is well described (*32*–*35*). These filter feeders become contaminated during growth or transport in sewage-contaminated water (*33*), unlike other food products that become contaminated by an infected food handler or cross-contamination. However, oysters were implicated as the vehicle of infection in <25% of the foodborne outbreaks, and a wide range of other vehicles were also reported. The greatest proportion of these outbreaks was attributed to ready-to-eat foods contaminated by infected food handlers. In the absence of a known zoonotic reservoir for NVs, the main reservoir of infection appears to be humans. Thus, reducing the incidence of foodborne NV infection requires interventions designed to prevent infected persons from contaminating prepared food and sewage from contaminating oyster beds.

These data, which show NV as the causative agent in 36% of outbreaks, support previous reports that NVs are the most common cause of infectious intestinal diseases in industrialized nations (*6*,*11*,*20*,*36*). NV accounts for a substantial extent of disease and potential economic loss, particularly to the health service where a large proportion of outbreaks occur. Wider consequences include ward closure, delayed discharge, and postponement of operations. Although NVs cause mild symptoms in healthy adults, the consequences of infection in vulnerable populations may be more serious. Considering that the populations of developed countries are aging, ensuring high levels of infection control in institutions caring for vulnerable groups is important.

## Conclusions

These analyses demonstrate the value of maintaining standardized outbreak surveillance over an extended period. By examining the epidemiologic characteristics of general outbreaks of NV by setting, we demonstrated that this pathogen is not merely an extremely common cause of infectious intestinal diseases but that its effects vary widely according to the population at risk. Within health-care institutions, NV contributes to substantial illness and is associated with substantial numbers of deaths. The elucidation of a distinct outbreak pattern that is characteristic of health-care institutions suggests that a combination of host, virologic, and environmental factors mediate these divergent epidemiologic patterns. Focused research studies need to be developed to investigate the population as well as the microbiologic and behavioral processes that might explain these observations. In addition, population-based studies incorporating virus typing are required to gain a deeper understanding of the epidemiology of sporadic NV infection in the wider population. Such studies are a prerequisite to the development of firm evidence-based and targeted control strategies.

## Appendix. Surveillance and analysis definitions

**Outbreak:** an incident in which two or more people, thought to have a common exposure, experience a similar illness or proven infection, at least one of them being ill (*22*).

**General outbreak**: an outbreak that affects members of more than one household, or residents of an institution (*36*).

**General outbreak of *Norovirus:*** a general outbreak in which *Nolovirus* is determined to be the causative agent by electron microscopy, RT-PCR, or enzyme immunoassay in one or more affected persons.

**Residential facilities:** includes residential homes, which provide some assistance in day-to-day living, and nursing homes, which provide care for persons whose infirmity or illness requires nursing care on a regular basis.

**Food outlets:** commercial food retailers including restaurants, pubs, bars, cafeterias, mobile food vendors, and caterers.
